# Coordination Modes of a Schiff Base Pentadentate Derivative of 4-Aminoantipyrine with Cobalt(II), Nickel(II) and Copper(II) Metal Ions: Synthesis, Spectroscopic and Antimicrobial Studies

**DOI:** 10.3390/molecules14010174

**Published:** 2009-01-01

**Authors:** Sulekh Chandra, Deepali Jain, Amit Kumar Sharma, Pratibha Sharma

**Affiliations:** 1 Department of Chemistry, Zakir Husain College (University of Delhi), J.L. Nehru Marg, New Delhi 110002, India; 2 Department of Chemistry, D.N. College, Meerut, India. E-mail: deepali101@yahoo.com (D. J.); 3 Division of Plant Pathology, IARI, Pusa, New Delhi, India. E-mail: psharma032003@yahoo.co.in (P. S.)

**Keywords:** 4-Aminoantipyrine derivative, Metal complexes, Biological activities.

## Abstract

Transition metal complexes of Co(II), Ni(II) and Cu(II) metal ions with general stoichiometry [M(L)X]X and [M(L)SO_4_], where M = Co(II), Ni(II) and Cu(II), L = 3,3’-thiodipropionic acid bis(4-amino-5-ethylimino-2,3-dimethyl-1-phenyl-3-pyrazoline) and X = NO_3_^−^, Cl^−^ and OAc^−^, have been synthesized and structurally characterized by elemental analyses, molar conductance measurements, magnetic susceptibility measurements and spectral techniques like IR, UV and EPR. The nickel(II) complexes were found to have octahedral geometry, whereas cobalt(II) and copper(II) complexes were of tetragonal geometry. The covalency factor (β) and orbital reduction factor (k) suggest the covalent nature of the complexes. The ligand and its complexes have been screened for their antifungal and antibacterial activities against three fungi, i.e. *Alternaria brassicae*, *Aspergillus niger* and *Fusarium oxysporum* and two bacteria, i.e. *Xanthomonas compestris* and *Pseudomonas aeruginosa*.

## Introduction

The transition metal complexes of 4-aminoantipyrine and its derivatives have been extensively examined due to their wide applications in various fields like biological, analytical and therapeutical [1,2,3,4]. Further, they have been investigated due to their diverse biological properties as antifungal, antibacterial, analgesic, sedative, antipyretic and anti-inflammatory agents [5,6,7]. In recent years, a number of research articles have been published on transition metal complexes derived from 4-amino-antipyrine derivatives with aza or aza-oxo donor atoms [8,9,10,11]. We were interested in examining the biological activities of NS-donor Schiff’s bases and their transition metal complexes, thus, in this article, we report the antifungal and antibacterial activities of the pentadentate (NNSNN-donor) Schiff’s base ligand 3,3’-thiodipropionic acid bis(4-amino-5-ethylimino-2,3-dimethyl-1-phenyl-3-pyrazoline) and its complexes with Co(II), Ni(II) and Cu(II) metal ions. The ligand and its complexes were characterized by physicochemical and spectral studies.

**Scheme 1:**
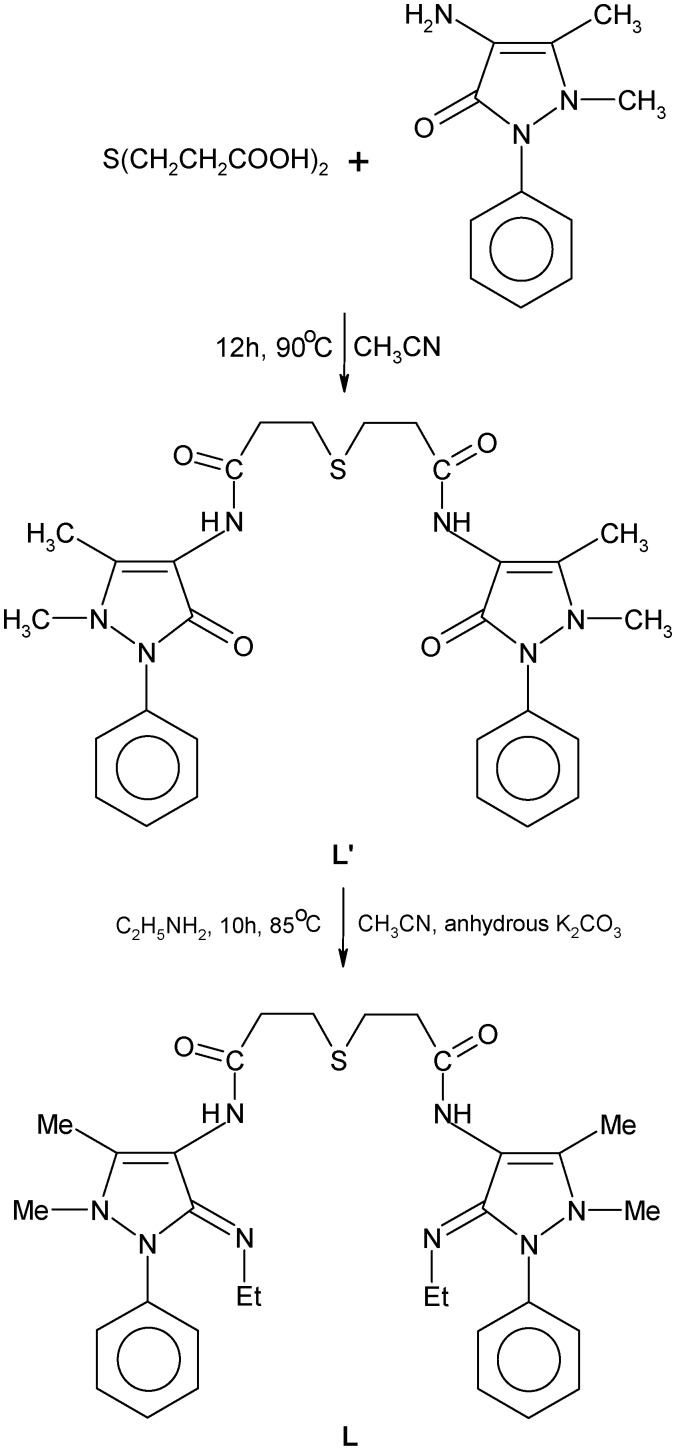
Synthesis of ligand.

## Results and Discussion

The synthesized novel Schiff’s base ligand, 3,3’-thiodipropionic acid bis(4-amino-5-ethylimino-2,3-dimethyl-1-phenyl-3-pyrazoline) (Scheme 1) forms stable complexes with Co(II), Ni(II) and Cu(II) metal ions. The analytical data of the complexes, together with their physical properties are given in Table 1. The analytical data of the complexes correspond to the general stoichiometry M(L)X_2_ and M(L)SO_4_ of the complexes, where M = Co(II), Ni(II) and Cu(II), L = ligand (C_32_H_42_N_8_SO_2_) and X = NO_3_^−^, Cl^−^ and OAc^−^. The value of molar conductance of complexes in DMSO indicates that the [M(L)SO_4_] complexes are non-electrolytes and [M(L)X]X are 1:1 electrolytes [12]. Magnetic moments lie in the range 5.01–5.08 B.M., 2.82–2.93 B.M. and 1.82–1.91 B.M. for Co(II), Ni(II) and Cu(II) complexes, respectively.

**Table 1:** Analytical data and physical properties of complexes.

S.No.	Complex	Color	m.p. (°C)	Molar conductance, (W^−1^cm^2^mol^−1^)	Yield (%)	Elemental analyses data (%) calculated (found)					
						M	C	H	N	S	Cl
1	[Co(L)NO_3_]NO_3_	Grey	272	112	54	7.50	48.92	5.35	17.84	4.08	-
	CoC_32_H_42_N_10_SO_8_					(7.45)	(48.86)	(5.30)	(17.79)	(4.03)	
2	[Co(L)Cl]Cl	Green	232	104	59	8.05	52.47	5.74	15.30	4.37	9.70
	CoC_32_H_42_N_8_SO_2_Cl_2_					(8.00)	(52.41)	(5.70)	(15.25)	(4.31)	(9.64)
3	[Co(L)OAc]OAc	Brown	238	98	58	7.56	55.46	6.16	14.38	4.11	-
	CoC_36_H_48_N_8_SO_6_					(7.51)	(55.41)	(6.10)	(14.33)	(4.06)	
4	[Co(L)SO_4_]	Green	222	15	49	7.78	50.73	5.55	14.80	8.46	-
	CoC_32_H_42_N_10_S_2_O_6_					(7.74)	(50.66)	(5.51)	(14.75)	(8.40)	
5	[Ni(L)NO_3_]NO_3_	Green	280	88	61	7.48	48.94	5.35	17.84	4.08	-
	NiC_32_H_42_N_10_SO_8_					(7.43)	(48.84)	(5.30)	(17.78)	(4.04)	
6	[Ni(L)Cl]Cl	Light Green	268	96	63	8.02	52.48	5.74	15.31	4.37	9.70
	NiC_32_H_42_N_8_SO_2_Cl_2_					(7.96)	(52.41)	(5.67)	(15.26)	(4.33)	(9.66)
7	[Ni(L)OAc]OAc	Light Green	276	106	60	7.54	55.48	6.16	14.38	4.11	-
	NiC_36_H_48_N_8_SO_6_					(7.47)	(55.42)	(6.10)	(14.33)	(4.04)	
8	[Ni(L)SO_4_]	Green	242	17	68	7.76	50.75	5.55	14.80	8.46	-
	NiC_32_H_42_N_10_S_2_O_6_					(7.71)	(50.68)	(5.50)	(14.76)	(8.38)	
9	[Cu(L)NO_3_]NO_3_	Green	190*	116	61	8.04	48.64	5.32	17.73	4.05	-
	CuC_32_H_42_N_10_SO_8_					(7.98)	(48.58)	(5.27)	(17.67)	(3.98)	
10	[Cu(L)Cl]Cl	Green	217	93	55	8.62	52.14	5.70	19.01	4.35	9.64
	CuC_32_H_42_N_8_SO_2_Cl_2_					(8.55)	(52.08)	(5.64)	(18.96)	(4.28)	(9.59)
11	[Cu(L)OAc]OAc	Parrot Green	204*	87	62	8.10	55.14	6.13	14.29	4.08	-
	CuC_36_H_48_N_8_SO_6_					(8.04)	(55.06)	(6.06)	(14.23)	(4.02)	
12	[Cu(L)SO_4_]	Dark Green	266	15	58	8.34	50.43	(5.51)	14.71	8.41	-
	CuC_32_H_42_N_10_S_2_O_6_					(8.28)	(50.36)	(5.46)	(14.65)	(8.36)	

*decomposition temperature

### Mass spectra

**Figure 1:**
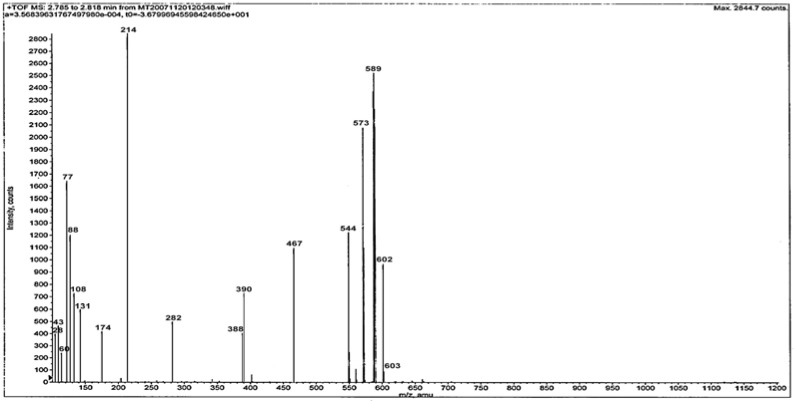
Mass spectrum of the ligand L.

Mass spectra provide a vital clue for elucidating the structure of compounds. The ESI mass spectrum of ligand L is given in Figure 1. The spectrum shows the molecular ion peak at m/z = 602 and the isotopic peak at m/z = 603 (M^+^+1) due to ^13^C and ^15^N isotopes. The base peak at m/z = 214 is due to the ethylimino-2,3-dimethyl-1-phenyl-3-pyrozoline (C_13_H_16_N_3_)^+^ ion. Another intense peak at m/z = 589 is due to a (C_31_H_39_N_8_SO_2_+2H)^+^ ion. The different competitive fragmentation pathways of ligand give the peaks at different mass numbers at 28, 29, 43, 60, 77, 88, 108, 131, 174, 282, 388, 390, 467, 544 and 573. The intensity of these peaks reflects the stability and abundance of the ions [13].

**Figure 2:**
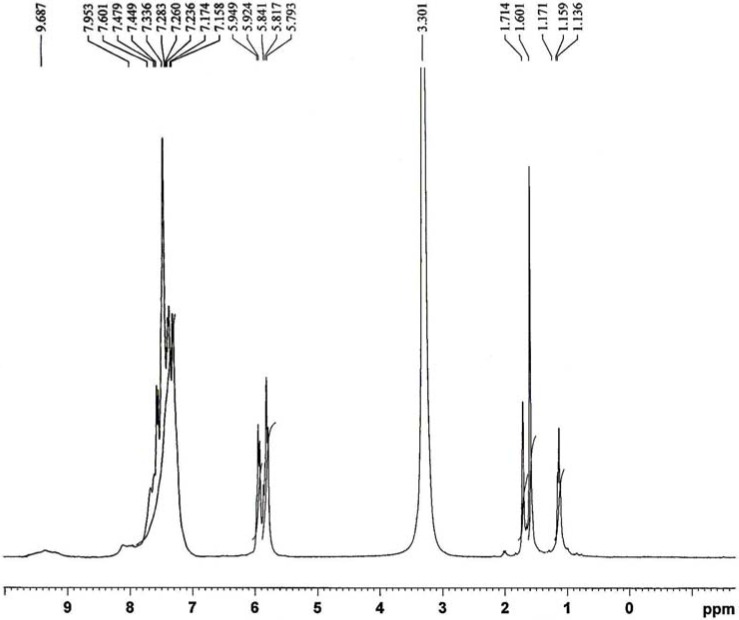
^1^H-NMR spectrum of the ligand L.

### NMR spectra

NMR data of the ligand are given in Table 2. Its ^1^H-NMR spectrum (Figure 2) displays a triplet at ca. δ 1.136-1.171 ppm (s, 6H, 2H_3_C-C), due to the six protons of two methyl groups attached to the CH_2_ groups, two singlets at ca. δ 1.601 ppm (s, 6H, 2H_3_C-C) and ca. δ 1.714 ppm (s, 6H, 2H_3_C-N) due to protons of methyl groups attached to the pyrazoline rings, two multiplets at ca. δ 5.7-5.9 ppm (m, 12H, 6CH_2_) due to the protons of six methylene groups and at ca. δ 7.1-7.9 ppm (m, 10H, aromatic) due to the protons of two phenyl rings and a broad signal at ca. δ 9.6 ppm (s, br, 2H, 2NH), corresponding to the two protons of two NH groups [14].

**Figure 3:**
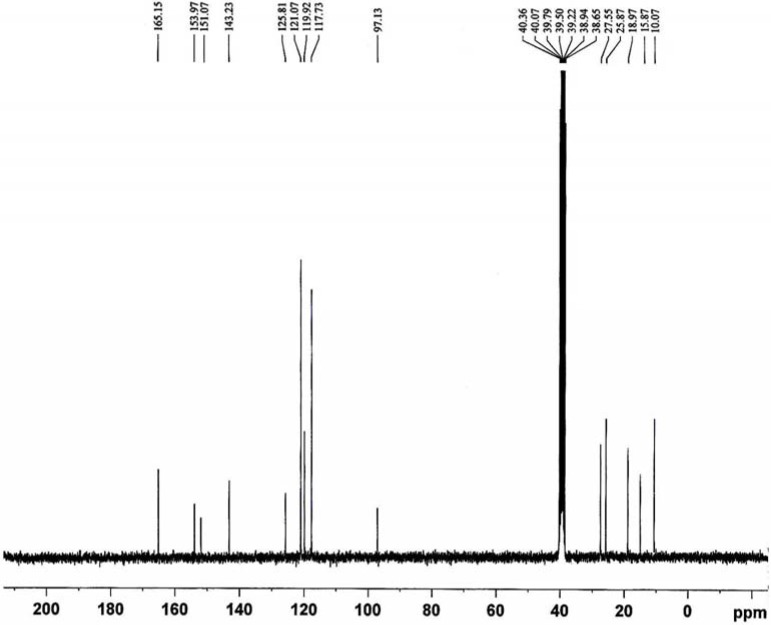
^13^C-NMR spectrum of the ligand L.

The ^13^C-NMR spectrum (Figure 3) displays the signals corresponding to the different non-equivalent carbon atoms at different values of δ as follows: at ca. δ 10.07 ppm (H_3_C-C), 15.87 ppm (H_3_C-H_2_C) and 18.97 ppm (H_3_C-N) corresponding to carbon atoms of methyl groups; at ca. δ 25.87 ppm (H_2_C-S), 27.55 ppm (H_2_C-N) and 97.13 ppm (MeH_2_C-N) due to methylene carbon atoms; at ca. δ 117.73, 119.92, 121.07 and 125.81 ppm due to the aromatic carbon atoms; at ca. δ 143.23 and 151.07 ppm (H_3_C-C and H_3_C-N) due to carbon atoms of pyrazoline rings; at ca. δ 153.97 ppm (C=N) due to carbon atoms azomethine groups and at ca. δ 165.15 ppm (C=O) due to carbon atom of carbonyl groups [15,16].

### IR spectra

Selected IR bands of the ligand and its complexes are listed in Table 3. The IR spectrum of the ligand displays bands at 1647, 1621 and 1532 cm^−1^, which may be assigned to the ν(C=O) amide I, ν(C=N) (azomethine linkage) stretching vibration and δ_NH_ (NH in-plane-bending) (amide III) vibrations. The band appearing at 768 cm^−1^ in the spectrum corresponds to the C-S stretching vibration. The C-S group is less polar in comparison to a C=O group and has a considerably weaker bond, so consequently the corresponding band appeared at a lower frequency. The bands corresponding to the C=N stretching, NH in-plane-bending and C-S stretching vibrations show the downward shift upon coordination which indicates that the nitrogen atoms of azomethine and NH groups and sulfur atom of C-S group are coordinated to the metal atom. However, the band corresponding to the C=O group (amide I) remains almost unchanged on complexation, which indicates that the carbonyl group oxygen atom is not involved in coordination. This discussion suggests that the ligand coordinates to metal atom in quinquedentate fashion (NNSNN) [17,18,19,20,21].

**Table 2:** The NMR data of the Schiff’s base ligand.

^1^H-NMR		^13^C-NMR	
δ (ppm)	Assignment	δ (ppm)	Assignment
1.136-1.171	t, 6H, 2H_3_C-H_2_C	10.07	C(7), C(20)
1.601	s, 6H, 2H_3_C-C	15.87	C(11), C(24)
1.714	s, 6H, 2H_3_C-N	18.97	C(5), C(21)
5.793-5.949	m, 12H, 6CH_2_	25.87	C(13), C(16)
7.158-7.953	m, 10H, Ar	27.55	C(14), C(15)
9.687	br, 2H, 2NH	97.13	C(10), C(23)
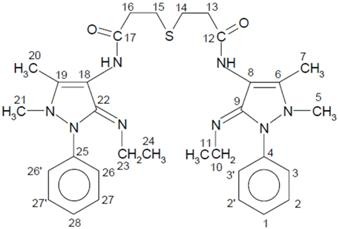		117.73	C(2, 2’), C(27, 27’)
		119.92	C(3, 3’), C(26, 26’)
		121.07	C(1), C(28)
		125.81	C(4), C(25)
		143.23	C(6), C(19)
		151.07	C(8), C(18)
		153.97	C(9), C(22)
		165.15	C(12), C(17)

The coordination behavior of the ligand is also verified by the appearance of new IR bands in the spectra of complexes in the 384-501 and 313-351 cm^−1^ range (Table 3). These bands may be assigned to ν(M-N) and ν(M-S) stretching vibrations, respectively. In addition, the IR spectra of complexes also display the bands due to anions. The nitrato complexes show the IR bands in the range 1397-1407 (ν_5_), 1304-1313 (ν_1_) and 1058-1075 cm^−1^ (ν_2_) due to NO stretching vibration of the NO_3_^−^ ion. The Δν i.e. ν_5_-ν_1_ (93-95 cm^−1^) indicates the unidentate coordination of NO_3_^−^ ion. The chloro complexes show the bands in the region 307-321 cm^−1^ to the ν(M-Cl). The acetato complexes give the IR bands in the region 1401-1412 cm^-1 ^and 1207-1214 cm^−1^ due to ν_as_(OAc) and ν_s_(OAc) stretching vibrations, respectively. The Δν i.e. 187-204 cm^−1^ suggests the unidentate coordination of OAc^−^ ion. In the sulphato complexes, the two medium intensity bands in the range 951-964 cm^−1^ (ν_1_) and 439-448 cm^−1^ (ν_2_) and a strong band 1037-1137 cm^−1^ (ν_3_) are appeared. The splitting of ν_3 _band in to two bands suggests the coordination of SO_4_^−2^ ion in unidentate manner [22]. 

**Table 3:** Selected IR bands of Schiff’s base ligand and its complexes.

Compound	ν(C=N)	ν(C=O) amide I	δ(NH) amide III	ν(C-S)	ν(M-N)	ν(M-S)	Anion bands
Ligand	1621s	1647vs	1532s	768ms	_	_	_
[Co(L)NO_3_]NO_3_	1570s	1648s	1494mw	761mw	425w	335w	1405mw, 1310m, 1058m
[Co(L)Cl]Cl	1601sh	1646s	1489m	740sh	464m	339m	307sh
[Co(L)OAc]OAc	1593m	1645s	1488m	717mw	403m	347sh	1412s, 1208m
[Co(L)SO_4_]	1576m	1748s	1501m	722m	436m	339mw	1087m, 1074m, 951m, 439mw
[Ni(L)NO_3_]NO_3_	1571ms	1651s	1493m	652br	479m	326m	1407m, 1313m, 1075mw
[Ni(L)Cl]Cl	1570m	1640m	1405m	673br	384sh	343sh	318sh
[Ni(L)OAc]OAc	1591m	1648ms	1406m	651br	475mw	329m	1406s, 1207s
[Ni(L)SO_4_]	1596m	1647s	1507m	668m	479m	313s	1048s, 1037s, 964m, 448m
[Cu(L)NO_3_]NO_3_	1576vs	1643s	1517s	669ms	422mw	342m	1397s, 1304s, 1064m
[Cu(L)Cl]Cl	1564w	1647s	1490m	699m	501w	351m	321sh
[Cu(L)OAc]OAc	1570vs	1650s	1492ms	684ms	477m	326m	1401m, 1214m
[Cu(L)SO_4_]	1568s	1648s	1490s	684m	458m	318sh	1137m, 1103m, 959m, 440mw

Abbreviations: vs = very strong, s = strong, ms = medium strong, m = medium, mw = medium weak, w = weak, br = broad, sh = sharp

### Electronic spectra

The electronic spectra of the complexes were recorded in DMSO solutions. The electronic spectral data of the complexes are given in Table 4. All the complexes show the high energy absorption band in the region 34,511–38,910 cm^−1^. This transition may be attributed to the charge transfer band.

The electronic spectra of cobalt(II) complexes display the d–d transition bands in the region 9,746–10,471, 15,247–19,493 and 18,621–22,371 cm^−1^. These transitions may be assigned to the ^4^T_1g _(F) → ^4^T_2g _(F) ν_1_, ^4^T_1g _(F) → ^4^A_2g_ (F) ν_2_ and ^4^T_1g_ (F) → ^4^T_1g_ (P) ν_3_, respectively. The transitions correspond to the tetragonal geometry of the complexes. 

The absorption spectra of nickel(II) complexes display three d-d transition bands in the range 11,135 –12,108, 18,621–19,416 and 21,413–27,322 cm^−1^. The transitions correspond to the ^3^A_2g _(F) → ^3^T_2g _(F) ν_1_, ^3^A_2g _(F) → ^3^T_1g_ (F) ν_2_ and ^3^A_2g_ (F) → ^3^T_1g_ (P) ν_3_, respectively. These transitions reveal that the nickel complexes possess an octahedral geometry and D_4h_ symmetry. 

Electronic spectra of copper(II) complexes show the d-d transition bands in the range 12,188–15,479, 18,621–19,132 and 24,402–27,322 cm^−1^. These bands correspond to ^2^B_1g_ ← ^2^A_1g_ (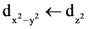) ν_1_,^2^B_1g_ ← ^2^B_2g_ (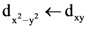) ν_2_ and ^2^B_1g_ ← ^2^E_g_ (
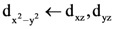
) ν_3_ transitions, respectively. The spectra are typical of Cu(II) complexes with an elongated tetragonal. The spectra of all the complexes have been vibronically assigned to D_4h_ symmetry with a 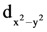 ground state. The most active vibration in this point group appears to be b_1u_ symmetry and its efficiency may arise from its being the only out-of-the-xy-plane vibration. The complexes are with one electron sequence i.e. 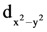 > 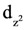 > d_xy_ > d_xz_, d_yz_ [23,24].

**Table 4:** Magnetic moment values, electronic spectral and ligand field parameters data of complexes.

Complex	m_eff_ (B.M.)	l_max_ (cm^−1^)	D_q_ (cm^−1^)	B(cm^−1^)	β	LFSE (kJmol^−1^)
[Co(L)NO_3_]NO_3_	5.02	10384, 16326,18621, 36101	984.2	532.03	0.48	94.07
[Co(L)Cl]Cl	5.08	9746, 19493, 22371, 37878	1186.9	913.10	0.81	113.44
[Co(L)OAc]OAc	5.06	10471, 16702, 21188, 36363	1177.1	784.74	0.70	112.50
[Co(L)SO_4_]	5.01	9856, 15247, 19555, 37724	1069.9	737.92	0.66	102.27
[Ni(L)NO_3_]NO_3_	2.84	11248, 18621, 21413, 36101	1124.8	419.33	0.40	161.26
[Ni(L)Cl]Cl	2.87	11185, 18688, 27322, 38022	1118.5	830.33	0.79	160.36
[Ni(L)OAc]OAc	2.93	11135, 18621, 25510, 36900	1113.5	715.07	0.69	159.64
[Ni(L)SO_4_]	2.82	12108, 19416, 26131, 37028	1210.8	614.86	0.59	173.59
[Cu(L)NO_3_]NO_3_	1.82	13042, 19066, 24402, 34511	-	-	-	-
[Cu(L)Cl]Cl	1.91	15479, 18621, 27322, 38910	-	-	-	-
[Cu(L)OAc]OAc	1.86	12188, 19132, 25382, 35610	-	-	-	-
[Cu(L)SO_4_]	1.85	14022, 18651, 26052, 36020	-	-	-	-

The ligand field parameters like Racah inter-electronic repulsion parameter B, ligand field splitting stabilization energy 10 Dq, covalency factor β and ligand field stabilization energy (LFSE) have been calculated for the Co(II) and Ni(II) complexes. The values of B and Dq of Co(II) complexes were calculated from the transition energy ratio diagram using ν_3_/ν_1_ ratio. The value of β for the complexes under study accounts for the covalent nature of the complexes. The evaluated parameters are listed in Table 4.

### EPR spectra

The X-band EPR spectra of the Co(II) complexes were recorded at liquid nitrogen temperature in polycrystalline form. The line shaped EPR spectra of Co(II) complexes with g_iso_ = 2.10−2.14 (Table 5) correspond to the tetragonal symmetry around the Co(II) atoms. 

As a consequence of the fast spin-relaxation time of high-spin cobalt(II) ion, the signals are observed only at low temperature. The polycrystalline powder EPR signals for the Co(II) complexes are broad. The spectra are consistent with an S = 3/2 spin state. No hyperfine splitting of the transitions is detected since it is difficult to resolve this splitting in nonmagnetically diluted Co(II) complexes. The line shapes are mostly dominated by the unresolved hyperfine interactions and by a distribution of E/D, where E and D describe the axial and rhombic Zero field splitting (ZFS) constants, respectively. The spread of E/D results in a spread of g-values (g-strain). The dominant broadening effect is realized when the g-strain is converted in <B-strain> with the relation ΔB = -(hν/β)(Δg/g^2^), where the parameters have their usual definitions. Thus, the largest and smallest g-values of the S = 3/2 spectrum have field widths that differ by an order of magnitude, rationalizing why the high field features of the spectra are so broad [25,26]. 

**Table 5:** EPR parameters and orbital reduction parameters of Co(II) and Cu(II) complexes.

Complex	g_⊥_	g_||_	g_iso_	G	k_⊥_^2^	k_||_^2^	k
[Co(L)NO_3_]NO_3_	2.0006	2.3694	2.12	-	-	-	-
[Co(L)Cl]Cl	2.0034	2.4128	2.14	-	-	-	-
[Co(L)OAc]OAc	2.0018	2.3917	2.13	-	-	-	-
[Co(L)SO_4_]	2.0021	2.2916	2.10	-	-	-	-
[Cu(L)NO_3_]NO_3_	2.0311	2.2402	2.10	8.26	0.42	0.69	0.74
[Cu(L)Cl]Cl	2.0319	2.2421	2.10	8.10	0.49	0.67	0.71
[Cu(L)OAc]OAc	2.0216	2.1839	2.08	9.41	0.30	0.53	0.61
[Cu(L)SO_4_]	2.0294	2.2112	2.09	7.71	0.43	0.59	0.69

**Figure 4:**
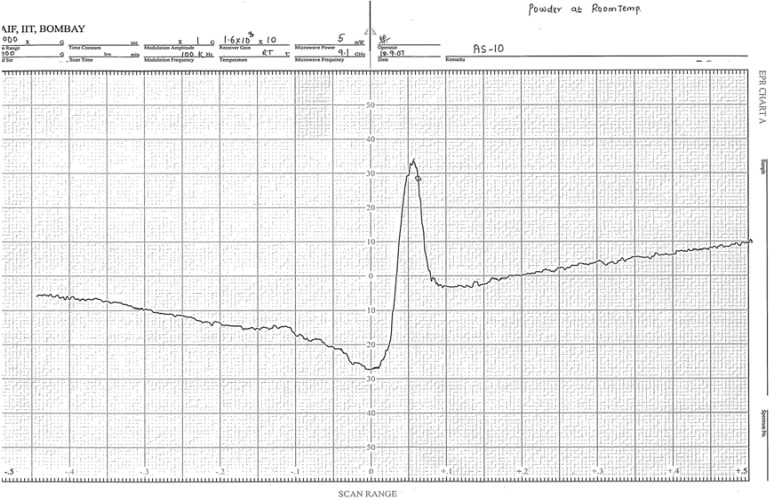
EPR spectrum of the [Cu(L)Cl]Cl complex.

The X-band EPR spectra of copper(II) complexes were recorded at room temperature in polycrystalline form. The spectra show only one broad signal at g_iso_ = 2.08 – 2.10 (Figure 4). The spectral studies reveal that the Cu(II) ion in the present complexes is in tetragonal field and shows the D_4h_ symmetry. The calculated values of g_||_ and g_⊥_ for the complexes show the order as g_||_ > g_⊥_ > 2.0023 (Table 5), which is consistent with the 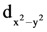 ground state [27,28]. The odd electron is located in the B_1g_ antibonding orbital.

The geometric parameter G i.e. the measurement of exchange interaction between the copper centres in the polycrystalline compounds, is calculated by using the expression:

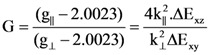


The complexes in the present study show the G values greater than 4 (Table 5), which suggest that the interaction between metal centres is negligible [24].

**Figure 5:**
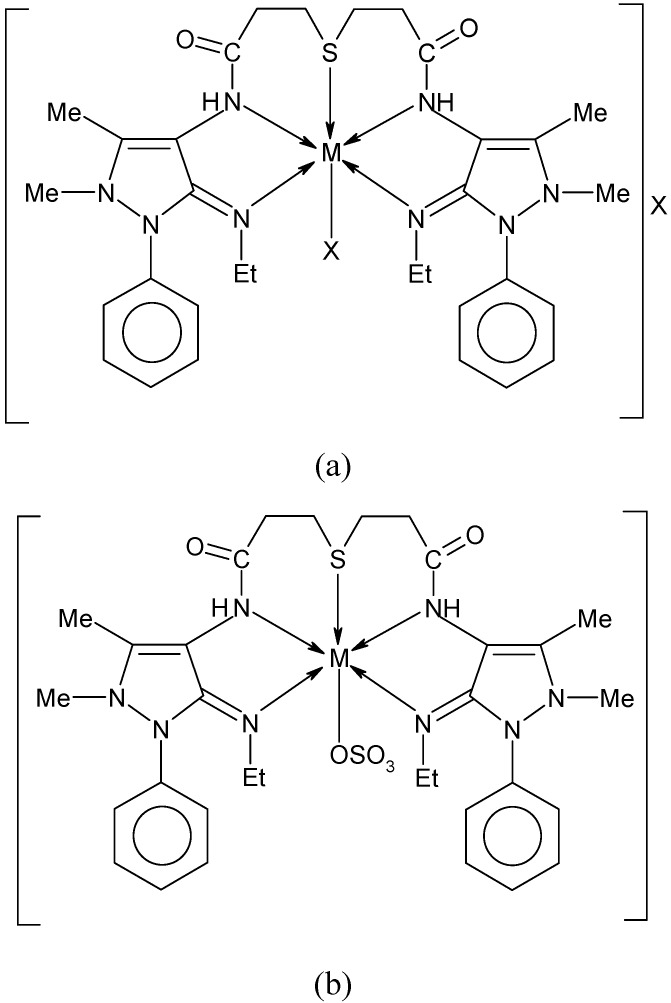
Structure of complexes (a) [M(L)X]X, (b) [M(L)SO_4_], where M =Co(II), Ni(II) and Cu(II), L = ligand and X = NO_3_^−^, Cl^−^ and OAc^−^.

For the copper(II) complexes, the EPR parameters and the d-d transition energies are used to evaluate, the orbital reduction factor k by using the expression: k^2^ = k_||_^2^ + 2k_⊥_^2^/3, where k_||_ and k_⊥_ are the parallel and perpendicular components of the orbital reduction factor. The low values of k (0.61-0.74) indicate the covalent nature of the complexes (Table 5). On the basis of above discussion, the structures of complexes are given in Figure 5.

### Antimicrobial activities

**Figure 6:**
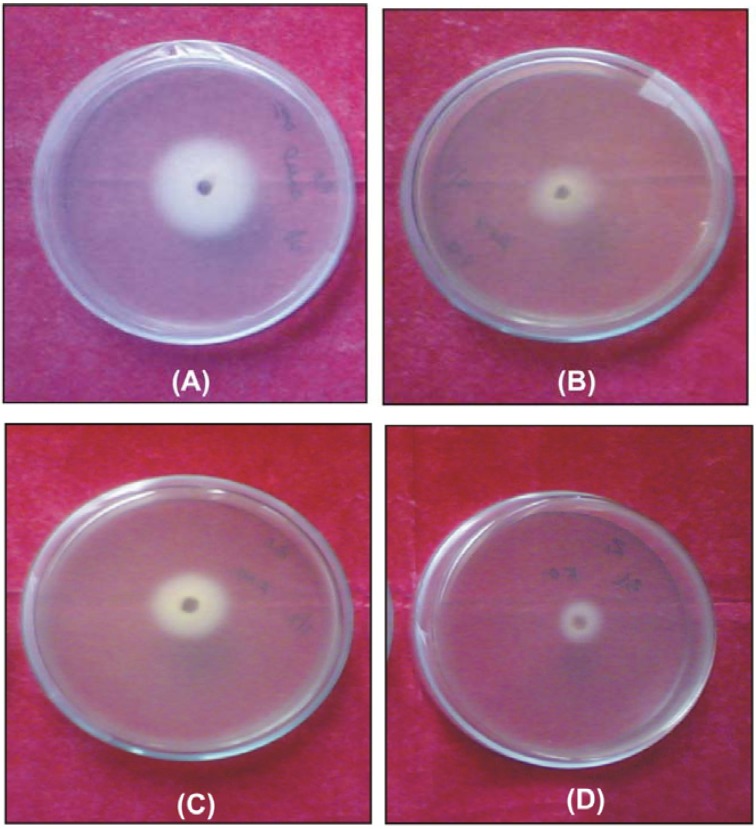
Antifungal activity against *Fusarium oxysporum* of: (A) ligand; (B) [Ni(L)NO_3_]NO_3_; (C) [Co(L)Cl]Cl and (D) [Cu(L)NO_3_]NO_3_.

The data of the antifungal and antibacterial activities of ligand and complexes are given in Table 6 and Table 7. The data reveal that the complexes have higher activities than the free ligand (Figure 6). This enhancement of the activity of ligand on complexation can be explained by Overtone’s Concept and Chelation Theory [29]. The theory states that chelation reduces the polarity of the metal atom by the partial sharing of its positive charge with donor groups and possible π–electron delocalization over the whole ring. This results with increasing of the lipophilic character of the complex and favor the permeation of the complex through the lipid layer of cell membrane. The complex blocks the metal binding sites in the enzymes of microorganisms. Consequently, the complex disturbs the metabolism pathways in cell and as a result microorganisms die.

**Table 6:** Antifungal activity data of compounds.

Compound	Fungal inhibition (%) (conc. in μg∙mL^−1^)								
	*A. brassicae*			*A. niger*			*F. oxysporum*		
	100	200	300	100	200	300	100	200	300
Ligand (L)	40	52	62	35	50	58	42	60	66
[Co(L)NO_3_]NO_3_	52	61	70	50	60	68	52	70	77
[Co(L)Cl]Cl	50	60	68	48	61	65	50	68	76
[Co(L)OAc]OAc	50	60	70	50	59	66	49	70	78
[Co(L)SO_4_]	48	59	68	50	60	66	50	70	76
[Ni(L)NO_3_]NO_3_	58	70	78	65	74	80	60	74	85
[Ni(L)Cl]Cl	58	68	75	62	75	82	58	75	86
[Ni(L)OAc]OAc	55	65	76	64	70	80	60	72	85
[Ni(L)SO_4_]	57	68	76	63	72	80	59	74	84
[Cu(L)NO_3_]NO_3_	60	72	80	64	75	84	60	74	90
[Cu(L)Cl]Cl	58	70	81	65	71	85	59	74	88
[Cu(L)OAc]OAc	60	71	80	65	70	82	60	72	90
[Cu(L)SO_3_]	60	72	80	65	74	85	60	72	88
Standard (Captan)	70	80	100	75	90	100	65	75	100

**Table 7:** Antibacterial activity data of compounds.

Compound	Bacterial inhibition zone (mm) (conc. in μg∙mL^−1^)					
	*Xanthomonas compestris*			*Pseudomonas aeruginosa*		
	250	500	1000	250	500	1000
Ligand (L)	10	12	15	8	12	14
[Co(L)NO_3_]NO_3_	14	16	21	15	18	20
[Co(L)Cl]Cl	14	16	20	14	18	20
[Co(L)OAc]OAc	14	17	19	14	17	20
[Co(L)SO_4_]	14	16	20	15	18	19
[Ni(L)NO_3_]NO_3_	16	20	25	17	22	25
[Ni(L)Cl]Cl	15	21	24	16	20	25
[Ni(L)OAc]OAc	15	20	24	16	20	24
[Ni(L)SO_4_]	16	20	24	16	21	25
[Cu(L)NO_3_]NO_3_	16	21	26	18	22	28
[Cu(L)Cl]Cl	16	22	25	18	23	27
[Cu(L)OAc]OAc	17	22	26	17	21	27
[Cu(L)SO_4_]	17	22	25	18	22	26

## Conclusions

The ligand 3,3’-thiodipropionic acid bis(4-amino-5-ethylimino-2,3-dimethyl-1-phenyl-3-pyrazoline, characterized on the basis of elemental analysis, IR, Mass, ^1^H-NMR and ^13^C-NMR spectral studies, coordinates to Co(II), Ni(II) and Cu(II metal ions in a pentadentate (NNSNN) manner. The value covalency factor (β) and orbital reduction factor (k) suggest the covalent nature of the complexes. The screening of biological activities of ligand and its complexes against the fungi *Alternaria brassicae*, *Aspergillus niger* and *Fusarium oxysporum* and the pathogenic bacteria *Xanthomonas compestris* and *Pseudomonas aeruginosa* indicates that the complexes show the enhanced activity in comparison to free ligand.

## Experimental

### General

3,3’-Thiodipropionic acid, 4-aminoantipyrine and ethylamine were obtained from Sigma-Aldrich and were of AR grade. Metal salts (E. Merck), other different chemicals (Fluka and Thomas Baker), sterile discs (Himedia) and solvents (S.D. Fine) were commercial products and were used as received. The stoichiometric analyses were carried out on a Carlo-Erba 1106 analyzer. Metal contents were estimated on an AA-640-13 Shimadzu flame atomic absorption spectrophotometer in solution prepared by decomposition of the complex in hot concentrated HNO_3_. The ^1^H-NMR spectrum was recorded with a model Bruker Advance DPX-300 spectrometer operating at 300 MHz using EtOD as a solvent and TMS as an internal standard. IR spectra were recorded as KBr pellets and CsI pellets (for chloro complexes) in the region 4,000-200 cm^−1^ on a FT-IR spectrum BX-II spectrophotometer. Electron spray ionization mass spectrum was recorded on a model Q Star XL LCMS-MS system at source temperature 300°C and voltage with +ve mode 5,500 V and –ve mode 4,500 V. The electronic spectra were recorded on Shimadzu UV mini-1240 spectrophotometer using DMSO as a solvent. EPR spectra were recorded for solids on an E4-EPR spectrometer at room temperature and liquid nitrogen temperature operating at X-band region with 100 KHz modulation frequency, 5 mw microwave power and 1 G modulation amplitude using DPPH as standard. The molar conductance of complexes was measured in DMSO at room temperature on an ELICO (CM 82T) conductivity bridge. The magnetic susceptibility was measured at room temperature on a Gouy balance using CuSO_4_.5H_2_O as callibrant.

### Synthesis of the Schiff’s base ligand, 3,3’-thiodipropionic acid bis(4-amino-5-ethylimino-2,3-dimethyl-1-phenyl-3-pyrazoline)*(**L**)*

The Schiff’s base ligand was synthesized in two steps:

1. *3,3’-Thiodipropionic acid bis(4-amino-2,3-dimethyl-1-phenyl-3-pyrazolin-5-one)* (**L’**)**:** A hot solution of 3,3’-thiodipropionic acid (0.02 mol, 3.5642 g) in acetonitrile (15 mL) was slowly added dropwise to a hot solution of 4-aminoantipyrine (0.04 mol, 8.13 g) in acetonitrile (30 mL). The resulting solution was refluxed for 12 h at 90°C, then allowed to cool and solvent was removed under reduced pressure. A light brown precipitate was obtained, which was separated out by absolute ethanol. The product was filtered, washed with cold ethanol and dried under vacuum over P_4_O_10_. Yield 54%, m.p. 210°C. Anal. calcd. for C_28_H_32_N_6_SO_4_: C, 61.31; H, 5.84; N, 15.33; S, 5.84. Found: C, 61.26; H, 5.81; N, 15.29; S, 5.79(%).
2. *3,3’-Thiodipropionic acid bis(4-amino-5-ethylimino-2,3-dimethyl-1-phenyl-3-pyrazoline)* (**L**): To the hot solution of 3,3’-thiodipropionic acid bis(4-amino-2,3-dimethyl-1-phenyl-3-pyrazolin-5-one) (0.01 mol, 5.48 g) in actonitrile (30 mL), a hot solution of ethylamine (0.02 mol, 1.11 mL) in acetonitrile (10 mL) was slowly added dropwise with constant stirring. The mixture was refluxed for 10 h at 85°C, allowed to cool at room temperature and the solvent was removed under reduced pressure. The resulting cream colored product was dissolved in absolute ethanol, filtered, washed with cold ethanol and dried under vacuum over P_4_O_10_. Yield 60%, m.p. 200°C. Anal. calcd. for C_32_H_42_N_8_SO_2_: C, 63.79; H, 6.98; N, 18.61; S, 5.32. Found: C, 63.73; H, 6.94; N, 18.56; S, 5.28(%).

### Synthesis of the complexes

To a hot solution of Schiff’s base ligand (1 mmol) in acetonitrile (15 mL), a hot solution of corresponding metal salt like nitrate, chloride, acetate or sulphate (1 mmol) in acetonitrile (10 mL) was added slowly with constant stirring. The resulting mixture was refluxed for 8–10 h at 70–80°C. On cooling the mixture overnight at 0°C, the colored product which separated out was filtered, washed with acetonitrile and dried under vacuum over P_4_O_10_.

### Biological activities

The Disc Diffusion Method and Food Poison Technique [30,31] were employed for screening the antibacterial and antifungal activities, respectively, of the ligand and its complexes. The compounds were screened for their antifungal and antibacterial properties using three fungi – *Alternaria brassicae*, *Aspergillus niger* and *Fusarium oxysporum* – and two bacteria – *Xanthomonas compestris* and *Pseudomonas aeruginosa*.

The antibacterial activity was determine with the Disc Diffusion Method. Stock solutions were prepared by dissolving the compounds in DMSO and serial dilutions of the compounds were prepared in sterile distilled water to determine the Minimum Inhibition Concentration (MIC). The nutrient agar medium was poured into Petri plates. A suspension of the tested microorganism (0.5 mL) was spread over the solid nutrient agar plates with the help of a spreader. Fifty μL of the stock solutions was applied on the 10 mm diameter sterile disc. After evaporating the solvent, the discs were placed on the inoculated plates. The Petri plates were sealed with Parafilm^®^ and first placed at low temperature for two hours to allow the diffusion of a chemical and then incubated at a suitable optimum temperature (29 ± 2°C) for 30-36 hours. The diameter of the inhibition zones was measured in millimeters. DMSO was used as control and streptomycin as a standard drug.

The Food Poison Technique was used to determine the antifungal activity of the compounds. The stock solution of the compound was directly mixed into the PDA (Potato Dextrose Agar) medium at the tested concentration. A disc of 5 mm of test fungal culture of a specific age growing on solid medium was then cut with a sterile cork borer and was placed at the center of the solid PDA plate with the help of inoculums’ needle. The plates were sealed with Parafilm^®^ and incubated at 29 ± 2°C for 7 days. DMSO was used as a control and Captan as a standard fungicide. The inhibition of the fungal growth expressed in percentage terms was determined from the growth in the test plate relative to the respective control plate as given below:

Inhibition (%) = (C-T) 100 / C

where C = diameter of fungal growth in the control plate and T = diameter of fungal growth in the test plate.
